# The Association Between Potential Nuclear Factor-Kappa B1 Gene Polymorphism rs28362491 and miR-206 Level in Patients With Acute Lymphoblastic Leukemia

**DOI:** 10.14740/wjon2762

**Published:** 2026-06-25

**Authors:** Isra Muradi, Jehad Alhmoud, Moath Al-Qaraleh, Maher Sughayer, Khalid Halahleh

**Affiliations:** a Medical Laboratory Sciences, Al-Ahliyya Amman University, Al-Salt, Jordan; b Department of Pathology, King Hussein Cancer Center, Amman, Jordan; c Department of Internal Medicine, Section of Medical Oncology Haematology, Adult Bone Marrow Transplantation and Cellular Therapy Program, King Hussein Cancer Center, Amman, Jordan

**Keywords:** miR-206, NF-κB1-94 ATTG polymorphism, Acute lymphoblastic leukemia, Hodgkin lymphoma

## Abstract

**Background:**

microRNAs (miRNAs) and the nuclear factor-kappa B1 (NF-κB1) signaling pathway play a critical role in leukemogenesis. The miR-206 and NF-κB1-94 ATTG polymorphism (rs28362491) have a potential impact on cancer progression and treatment response. The primary objective of this study was to evaluate the levels of expression of miR-206, the genotypic distribution of the NF-κB1-94 ATTG polymorphism, and the secondary objective was to assess their relationship in patients with acute lymphoblastic leukemia (ALL), Hodgkin lymphoma (HL), and healthy controls.

**Methods:**

This was a retrospective case-control study conducted at King Hussein Cancer Center, from April to July 2023 and involved three distinct groups, ALL (n = 46), HL (n = 35), and healthy individuals (n = 30). Tissue samples were collected from patients, while blood samples from the control group. Data were retrieved from electronic medical records. The samples were analyzed for miR-206 expression and NF-κB1-94 ATTG polymorphism genotypes. miR-206 levels were measured using quantitative real-time polymerase chain reaction (qRT-PCR). Genotypic distributions were determined through PCR and subsequent sequencing. Statistical analyses evaluated correlations between miR-206 levels, NF-κB1 genotypes, and clinical outcomes.

**Results:**

miR-206 expression was significantly lower in ALL patients compared to healthy controls (P < 0.0001), with mean values of 0.01 ± 0.1 in ALL, 0.01 ± 0.02 in HL, and 2,777.2 ± 31.55 in control subjects, suggesting a potential tumor suppressor role in ALL and HL. The genotypic distribution of the NF-κB1 (rs28362491) polymorphism revealed that homozygous Ins/Ins genotype was most prevalent in ALL compared with controls (41.3% vs 12%, P = 0.0048), and lowest in HL (28.5%). The heterozygous Ins/Del genotype was most prevalent in controls (60%), HL (42.8%), and least common in ALL (30.4%), indicating a possible protective effect against ALL. Further analysis showed no significant differences in miR-206 levels across the NF-κB1 genotypes (P = 0.9086) according to remission status for heterozygous Ins/Del and homozygous Del/Del compared to homozygous Ins/Ins.

**Conclusions:**

This study suggests that: 1) there was no significant difference in miR-206 expression across different NF-κB1-94 ATTG polymorphism genotypes; 2) reduced miR-206 expression could have a potential tumor suppressor role in ALL patients; 3) there was no significant genotype effect on remission status in ALL patients; and 4) both abnormalities could serve as biomarkers.

## Introduction

Cancer is characterized by clonal expansion and genetic aberrations and involves complex molecular mechanisms that promote uncontrolled cell growth and survival. Hematologic malignancies, such as acute lymphoblastic leukemia (ALL), are particularly influenced by genetic and epigenetic factors, including microRNAs (miRNAs) and the nuclear factor-kappa B1 (NF-κB1) signaling pathways [1–7]. Despite advances in understanding these mechanisms, the interplay between miR-206 levels, NF-κB1 (rs28362491) polymorphism, and their impact on ALL remains unclear. This gap hinders the development of targeted therapies and effective prognostic tools [6, 7].

Several miRNAs have been identified as novel biomarkers for prognostic stratification in ALL, and NF-κB1 gene plays a major role in various types of leukemia [8–17]. In ALL, the most frequently altered miRNAs include the miRNA-181 cluster, which is considered a crucial oncomimetic factor in childhood ALL [8]; miRNA-155, which induces pre-B cells clonal expansion and is overexpressed in different pediatric ALL subtypes [9, 10]; and miRNA-128b, which allows differentiation from acute myeloid leukemia (AML) cases and is downregulated in ALL with the MLL-AF4 translocation [11, 12]. Other specific genes in several types of leukemia were detailed in Table 1 [3].

**Table 1:** miRNAs and Their Expression in ALL

ALL	miRNAs	Expression data
MLL rearranged	MiR-1, let-7b28b, miR-708	Downregulation
t (12;21) (p13; q22) ETV6-RUNX1	MiR-100, miR-125b, miR-99a, miR-126, let-7c, miR-181a	Upregulation of miR-100, miR-125b, miR-99a, miR-126, let-7c; downregulation of miR-181a
t (9;22) (q34; q11) *BCR-ABL1*	MiR-125b, miR-203	Overexpression of miR-125b; downregulation of miR-203
Hyperdiploid karyotype	MiR-222, miR-223, miR-374, miR-660, miR-98 and miR-511	Upregulation
t (1;19) (q23; p13) TCF3-PBX1	MiR-126, miR-146a, miR-511, miR-545, miR-365, miR-24, miR-30d, miR-193, miR-181, miR-708	Downregulation of miR-126, miR-146a, miR-511, miR-545, miR-365, miR-24, miR-30d, miR-193; upregulation of miR-181, miR-708
T-ALL	MiR-17-92, miR-128, miR-708, miR-196b, miR-128, miR-181, miR-29, miR-150, miR-99a, and miR-708	Overexpression of miR-17-92, miR-708, miR-196b, miR-128, miR181; downregulation of miR-29

miRNAs: microRNAs; ALL: acute lymphoblastic leukemia; MLL: mixed lineage leukemia; *BCR-ABL1*: breakpoint cluster region–Abelson murine leukemia viral oncogene homolog 1.

There are two well-known NF-κB1 pathways, both of which have been reported in several types of human hematological malignancies, mainly lymphoid leukemia and lymphoma [13–15]. The deletion of ATTG polymorphism (rs28362491) in the NF-κB1 gene promoter functions is a key regulator of NF-κB1 signaling, with the deletion (Del) allele leading to reduced transcription of the NF-κB1 gene, which impacts downstream inflammatory responses and may modulate expression patterns of miRNAs, including miR-206, through the regulation of transcription factor networks involved in differentiation and proliferation [13–15].

This study aims to evaluate the expression levels of miR-206 in ALL patients and healthy controls, investigate the genotypic distribution of the NF-κB1-94 ATTG polymorphism in patients with ALL, HL patients, and controls, explore the relationship between miR-206 levels and NF-kB1 genotypes in ALL patients, and assess the potential of miR-206 and NF-κB1 polymorphism as biomarkers for ALL diagnosis, prognosis, and therapeutic response.

## Materials and Methods

### Study design

This is a retrospective case-control study not involving human subjects and was conducted in accordance with the Declaration of Helsinki. Ethical approval was obtained from the Scientific Institutional Review Board (IRB) of the Faculty of Allied Medical Sciences at Al-Ahliyya Amman University (AAU) and the King Hussein Cancer Center (KHCC) (IRB number: 23KHCC39) before the commencement of the study. Informed consent was waived by the IRB of the KHCC due to the retrospective nature of the study. The materials and tissue samples used in the study were collected from paraffin-embedded bone marrow biopsy blocks obtained from ALL patients, lymph nodes from Hodgkin lymphoma patients at diagnosis, and blood samples from healthy controls, as summarized in Table 2. Study procedures were detailed here (Supplementary Material 1, wjon.elmerpub.com). Participants included three distinct groups of participants, 46 patients with ALL, 35 patients with HL, and a healthy control group of 30 healthy individuals. The research was conducted from April to July 2023 at the Hematology and Oncology Clinics of the KHCC. All baseline laboratory investigations were performed in the Laboratory Department of the KHCC. Data were retrieved from the patient’s electronic medical records to ensure comprehensive and accurate information. Advanced molecular tests were conducted at the Pharmacological and Diagnostic Research Center (PDRC) at AAU, utilizing state-of-the-art facilities and expertise to ensure thorough analysis and validation of the collected samples.

**Table 2:** Materials Used in the Study

Kit	Supplier	Catalog No.
miRNeasy FFPE Advanced Kit	Qiagen, Germany	No 217504
miRCURY LNA RT Kit	Qiagen, Germany	No. 339340
Quick-DNA/RNAm™ PFPE Kit	ZYMO Research, USA	No 3067
miRCURY LNA SYBR^®^ Green PCR Kit (600)	Qiagen, Germany	No. 339346
TaqMan^®^ Universal PCR Master	Thermo Fisher Scientific, USA	No. 4371355

PCR: polymerase chain reaction.

The selection criteria for participants in this study were strictly defined to ensure a homogeneous and relevant sample. The inclusion criteria included patients who were diagnosed with ALL or HL and undergoing treatment at KHCC during the study period. Both female and male adults aged 18 years or older were eligible. Conversely, the exclusion criteria were applied to maintain the integrity of the study’s focus and data quality. Patients who did not have a diagnosis of ALL, HL, or those with missing demographic data were excluded from participation. This approach ensured that the study results would accurately reflect the characteristics and treatment outcomes of the targeted patient population. All tissue samples were collected at the Hematology and Oncology Clinics of the KHCC, and blood samples were collected from the healthy control group, followed by miR-206 extraction and isolation. Plasma and tissue levels of miRNA were quantified using quantitative real-time polymerase chain reaction (qRT-PCR) with Fast SYBR Green. DNA was extracted for NF-κB single-nucleotide polymorphism (SNP) analysis and quantified by qRT-PCR. We acknowledge the fact of several methodological and biological differences between tissue-derived and plasma-derived miRNA analyses, including differences in extraction methods, measurements, stability, extraction efficiency, and normal tissue/blood samples due to variations in tumor cellularity, fibrosis, and RNase activity. miRNA extraction from HL patients, particularly from formalin-fixed paraffin-embedded (FFPE) tissues, is notoriously difficult due to the low percentage of Reed–Sternberg cells within a high inflammatory background, leading to potential contamination from reactive immune cells. However, specific methods designed for FFPE tissues, including the use of sodium dodecyl sulfate (SDS) instead of conventional methods or optimized proteinase K digestion, can successfully stabilize and retrieve long miRNA transcripts. Samples from ALL patients are usually rich in blasts and generally offer better stability when collected properly in miRNA-stabilizing reagents (e.g., PAXgene) compared to solid tumor FFPE samples, as they avoid the extensive cross-linking caused by fixation. miRNA from tissue or peripheral blood samples of normal individuals is highly unstable and may degrade within minutes to hours if not immediately stabilized because of high RNases activity. Therefore, specialized stabilizing reagents, such as PAXgene or Tempus tubes, are required to prevent degradation, and high yields of miRNA can be obtained if appropriate stabilization methods are used, thereby preserving miRNA integrity during handling and storage.

### Statistical section

Data were expressed either as mean ± standard deviation (SD) or as numbers and percentages, as indicated. The allelic and genotype frequencies of the NF-κB1 insertion/deletion ATTG polymorphism were obtained by direct counting. Fisher’s exact test was used to compare genotype and allele frequencies between patients with ALL and controls (patients with HL). Odds ratios (OR) and 95% confidence intervals (CIs) were reported to evaluate the effects of differences in allelic and genotype distributions.

For the comparison between ALL patients and controls, an unpaired Student’s *t*-test was used to compare means. A one-way analysis of variance (ANOVA) followed by Tukey’s *post-hoc* test was employed for comparing parameters across different genotypes. A two-sided P value of < 0.05 was considered statistically significant, while a P value of < 0.001 was considered highly significant. Demographic comparisons between ALL patients and patients with HL were performed using the Chi-square test. This comprehensive statistical approach ensured robust analysis and interpretation of the data, facilitating the identification of significant associations and differences relevant to the study. All descriptive statistics, data analyses, and graphics were performed using GraphPad Prism version 9 (GraphPad Software, San Diego).

## Results

Patients and disease characteristics were detailed in Tables 3 and 4. The study included 46 patients with ALL, 35 patients with HL, and 30 healthy controls. Among the ALL patients, 21.7% were females, while 78.2% were males. In contrast, HL group had 57.1% females and 42.8% males, and the control group had 58.0% females and 38.7% males. A Chi-square test indicated significant differences in gender distribution between the ALL and the HL groups (P = 0.000725) and between the ALL and the control groups (P = 0.001081). However, there was no significant difference in gender distribution between the HL group and the control group (P = 0.815739) (Table 5). Gender can influence both disease susceptibility and certain miRNA expression profiles. The lack of gender matching between groups might raise a potential bias.

**Table 3:** Patients Disease Characteristics for Hodgkin Lymphoma

	Gender	Age (year)	WBC count ≥ 15,000/mm^3^ (1 point)	Lymphocyte count < 600/mm^3^ or < 8% of WBC count (1 point)	Disease status at last encounter	Patient’s status at last encounter	Relapse
1	Male	33	7	35%	CR	Alive	No
2	Male	34	14	26%	CR	Alive	No
3	Female	22	7	20%	Missed	Alive	Missed
4	Male	28	8	5.50%	CR	Alive	Yes
5	Male	36	10	20%	PR	Alive	Yes
6	Female	40	8	2.50%	CR	Alive	No
7	Male	38	10	51%	CR	Alive	No
8	Male	28	8	36%	PR	Alive	Yes
9	Male	28	10	20%	CR	Alive	Yes
10	Male	40	8	40%	CR	Alive	No
11	Female	36	9	30%	Missed	Alive	No
12	Female	50	Missed	Missed	CR	Alive	Yes
13	Male	22	9	35%	CR	Alive	Missed
14	Female	36	10	35%	PR	Alive	Yes
15	Female	25	9	34%	CR	Alive	No
16	Male	35	11	23%	Missed	Alive	Yes
17	Female	21	12	34%	NoCR	Alive	Yes
18	Male	24	13	23%	CR	Alive	No
19	Male	26	13	20%	VGPR	Alive	Yes
20	Female	22	12	45%	NoCR	Alive	Yes
21	Female	49	10	29%	PR	Alive	Yes
22	Female	41	23	32%	CR	Alive	No
23	Male	26	18	32%	CR	Alive	No
24	Male	36	10	21%	CR	Alive	No
25	Male	46	20	23%	CR	Alive	No
26	Male	25	10	34%	CR	Alive	Missed
27	Male	33	14	23%	CR	Alive	Missed
28	Male	23	10	34%	CR	Alive	Missed

WBC: white blood cell; CR: complete remission; NoCR: non-remission; PR: partial remission; VGPR: very good partial remission.

**Table 4:** Patients Disease Characteristics for Acute Lymphoblastic Leukemia

Number	Gender	Age at diagnosis (year)	WBC (× 10^9^/L)	Conventional karyotyping	PCR for *BCR-Abl*: positive/negative
1	Male	45	9.60	46,XY	Negative
2	Male	36	13.00	46,XY	Negative
3	Male	63	25.00	46,XY	Negative
4	Male	51	159.00	46,XY, t(9;22)	Positive
5	Male	21	7.00	46,XY	Negative
6	Female	20	61.00	46,XX,t(4;11)	Negative
7	Male	32	4.20	46,XY, t(9;22)	Positive
8	Male	22	0.50	46,XY	Negative
9	Male	35	60.00	46,XY	Negative
10	Male	44	9.50	46,XY	Negative
11	Female	26	7.50	46,XY	Negative
12	Male	41	4.40	46,XY	Negative
13	Male	34	4.20	46,XY	Positive
14	Male	42	70.00	46,XY	Negative
15	Male	47	22.00	46,XY,t(4; 11)	Negative
16	Male	37	2.80	46,XY,t(9;22)	Positive
17	Male	36	61.00	47,XY,+21,der (1)(q), t(11;22)(p11;p11)	Negative
18	Male	35	60.00	46,XY	Negative
19	Male	21	5.80	47,XY, add,9,11	Negative
20	Male	18	22.00	46,XY	Negative
21	Male	24	18.00	46,XY	Negative
22	Female	51	2.10	46,XY	Negative
23	Female	21	37.00	Missed	Negative
24	Male	20	272.00	46,XY	Negative
25	Female	51	2.00	46,XX	Negative
26	Female	54	66.00	46,XX	Negative
27	Male	31	160.00	Missed	Negative
28	Male	21	42.00	Missed	Positive
29	Female	28	75.00	46,XX,t(1;9)	Negative
30	Male	36	2.60	Missed	Positive
31	Female	38	8.20	Missed	Positive
32	Male	43	44.00	46,XY,t(9;22)	Positive
33	Female	51	11.00	46,XY,t(9;22),del7	Positive
34	Male	39	4.40	46,XY	Negative
35	Male	20	450	46,XY	Positive
36	Male	22	35.00	46,XY	Negative
37	Male	20	117.00	Missed	Negative
38	Male	37	12.00	Missed	Negative
39	Male	22	33.00	Missed	Negative
40	Male	25	13.00	Missed	Negative
41	Male	36	10.00	46,XY	Positive
42	Male	19	43.00	Missed	Negative
43	Male	28	25.00	Missed	Negative
44	Female	38	8.20	46,XY,t(9;22)	Negative
45	Male	46	22.00	46,XX,t(4;11),inv(14)	Negative
46	Male	41	70.00	46,XY	Negative

PCR: polymerase chain reaction; WBC: white blood cell; M: male; F: female; *BCR-ABL*: breakpoint cluster region–Abelson murine leukemia viral oncogene homolog.

**Table 5:** Gender Distribution Among the Participants

Gender	ALL (n = 46)	Hodgkin lymphoma (n = 35)	Control (n = 30)	χ^2^ test		
				P^a^ value	P^b^ value	P^c^ value
F, n (%)	10 (21.7%)	20 (57.1%)	18 (58%)	0.000725	0.815739	0.001081
M, n (%)	36 (78.3%)	15 (42.9%)	12 (42%)			

P^a^: control and ALL. P^b^: control and Hodgkin lymphoma. P^c^: ALL and Hodgkin lymphoma. M: male; F: female; ALL: acute lymphoblastic leukemia.

**Figure 1:**
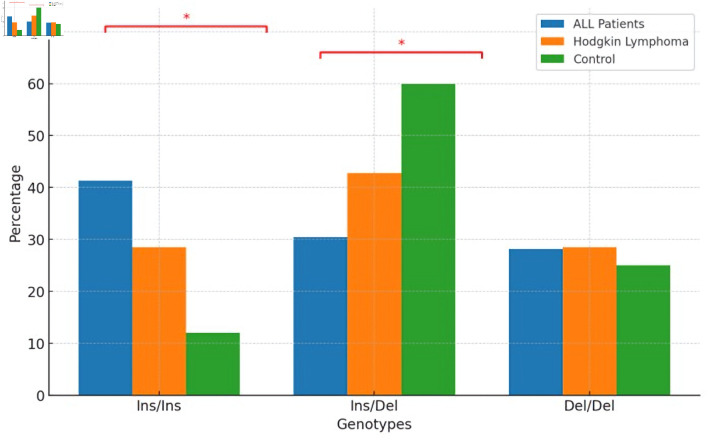
Distribution of NF-κB1 genotypes among different subject groups. NF-κB1: nuclear factor-kappa B1; ALL: acute lymphoblastic leukemia.

The genotypic distribution of the NF-κB1 (rs28362491) polymorphism was analyzed among the three groups. In the ALL-patient group, 41.3% were homozygous for the Ins/Ins genotype, 30.4% heterozygous for the Ins/Del genotype, and 28.2% were homozygous for the Del/Del genotype. In HL group, 28.5% were homozygous for the Ins/Ins genotype, 42.8% heterozygous for the Ins/Del genotype, and 28.5% were homozygous for the Del/Del genotype. Among the control group, 12% were homozygous for the Ins/Ins genotype, 60% heterozygous for the Ins/Del genotype, and 25% were homozygous for the Del/Del genotype (Fig. 1). The Chi-square test of independence initially showed no statistically significant difference in the overall distribution of NF κB1 genotypes across the three groups (P = 0.088). This suggested no statistically significant overall differences in genotype frequencies across the study groups.

To further investigate the relationships between specific groups, pairwise comparisons were conducted using Fisher’s exact test due to small sample sizes in some categories. The pairwise comparisons provided the following findings: between ALL patients and control subjects, there was a statistically significant difference in the Ins/Ins genotype (P = 0.015), indicating a higher prevalence of the Ins/Ins genotype in ALL patients compared to controls. Similarly, there was a statistically significant difference in the Ins/Del genotype (P = 0.023), suggesting a lower prevalence of the Ins/Del genotype in ALL patients compared to controls. There was no statistically significant difference observed for the Del/Del genotype between ALL patients and controls (P = 1.000), indicating similar frequencies of the Del/Del genotype in both groups (Fig. 1).

When comparing ALL patients with HL patients, no statistically significant differences were found for any of the genotypes (Ins/Ins, Ins/Del, Del/Del) (P = 0.254, P = 0.350, and P = 1.000, respectively), although the study may have lacked sufficient statistical power to detect meaningful differences. This suggests that genotype distributions are similar between ALL and HL groups. Similarly, no statistically significant differences were found in genotype distribution between control subjects and HL patients (P = 0.204, P = 0.295, and P = 1.000, respectively). This suggests that the genotype distributions are comparable between control subjects and patients with HL (Fig. 1).

The statistically significant differences in the Ins/Ins and Ins/Del genotypes between ALL patients and control subjects indicate potential genetic distinctions that may relate to disease susceptibility or therapy resistance. The higher prevalence of the Ins/Ins genotype and the lower prevalence of the Ins/Del genotype in ALL patients compared to controls suggest specific genetic profiles associated with ALL. The absence of significant differences in genotype distribution between ALL and HL patients, as well as between control and HL patients, suggests that these genotypes might not be influential factors in HL compared to ALL.

### miR-206 expression and clinical characteristics

**Figure 2:**
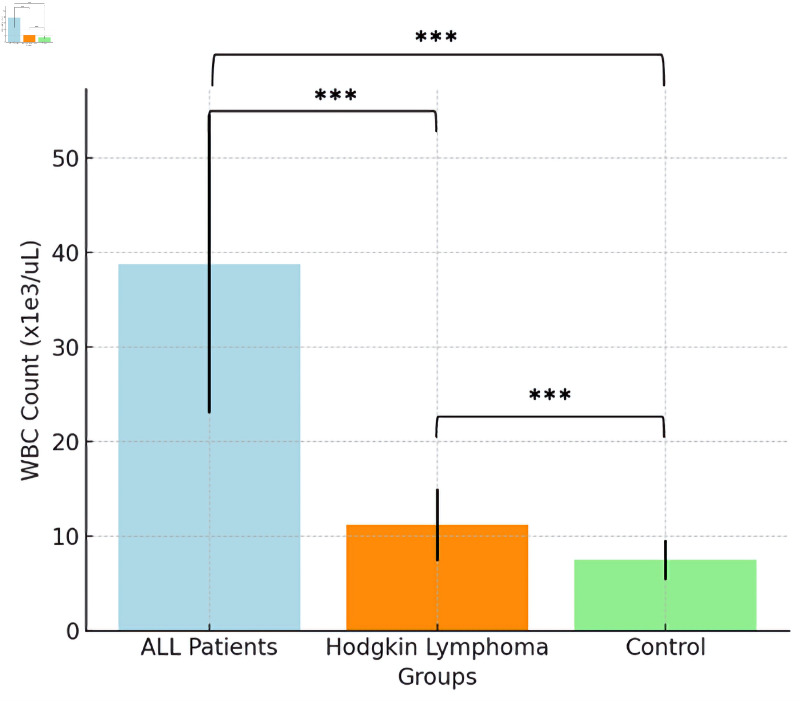
WBC counts across different subject groups. WBC: white blood cell; ALL: acute lymphoblastic leukemia.

This study analyzed white blood cell (WBC) counts and miR-206 expression levels across three distinct groups of ALL, HL patients, and control subjects. Data were presented as mean ± SD and were analyzed for statistical significance between groups. As shown in Figure 2, the mean WBC count for ALL patients was significantly higher (38.8 ± 15.8 × 10^3^/µL) compared to both HL patients (11.2 ± 3.8 × 10^3^/µL) and control subjects (7.5 ± 2.1 × 10^3^/µL). The differences between ALL patients and each of the other two groups were highly statistically significant (P < 0.0001). Additionally, the comparison between HL patients and control subjects revealed a statistically significant difference (P < 0.0001). These significantly elevated WBC counts in ALL patients likely indicate an acute immune response or the proliferation characteristic of leukemic conditions. In contrast, the lower WBC counts in HL patients compared to those with ALL may reflect different pathophysiological processes or treatment responses between these entities.

**Figure 3:**
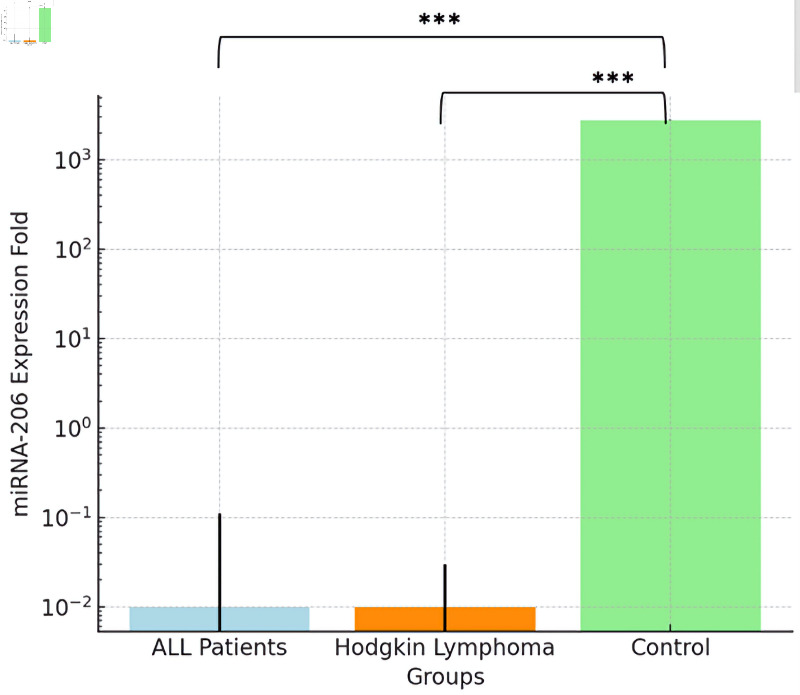
miR-206 expression across different subject groups. ALL: acute lymphoblastic leukemia; miRNA: microRNA.

miR-206 expression was markedly lower in both patient groups compared to controls. The mean values were close to baseline in ALL (0.01 ± 0.1) and HL patients (0.01 ± 0.02), whereas control subjects exhibited markedly elevated levels (2,777.2 ± 31.55). Significant differences in miR-206 expression were observed between the patient and the control groups (P < 0.0001). However, the difference between the ALL and HL patient groups was not significant (Fig. 3). The statistically significant difference in miR-206 expression between the control and the patient groups indicates a possible tumor suppressor role for miR-206, which appears to be reduced in ALL and HL. This hypothesis is supported by the absence of statistically significant differences in miR-206 expression between the two patient groups.

### Genotype and allele frequencies of the -94 ATTG polymorphism (rs28362491)

**Figure 4:**
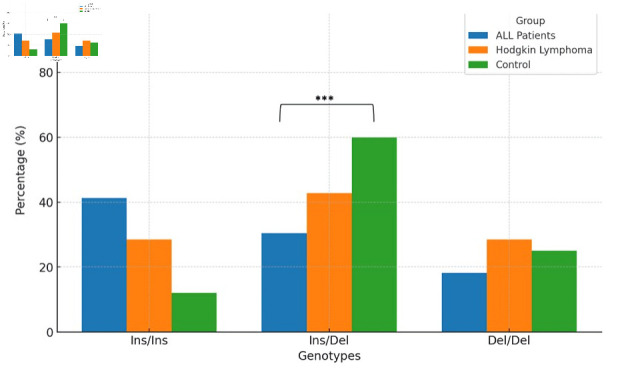
Genotype frequencies of -94 ATTG polymorphism (rs28362491) across different groups. ALL: acute lymphoblastic leukemia.

This study investigated the genotype and allele frequencies of the -94 ATTG polymorphism (rs28362491) across three distinct groups of ALL patients, HL patients, and control subjects. The frequencies were calculated as percentages of the total individuals in each group and were analyzed for statistical significance. While the genotype analysis provides a closer look at the potential genetic risks or protections, the allele frequency data offer insights into the broader genetic variability within and between populations. The frequency of the homozygous Ins/Ins genotype was highest in ALL patients (41.3%), followed by HL patients (28.5%), and was lowest in control subjects (12%). A statistically significant difference was observed between ALL patients and control subjects (P = 0.0048), indicating that this genotype is more prevalent among ALL patients (Fig. 4).

The heterozygous Ins/Del genotype was most prevalent in the control group (60%), followed by HL patients (42.8%), and ALL patients (30.4%). Statistically significant differences were found between ALL patients and control subjects, highlighting a distinctive distribution pattern that may suggest a protective effect or differing genetic predispositions. The frequencies of the homozygous Del/Del genotype were relatively consistent across all groups, with 28.2% in ALL patients, 28.5% in HL patients, and 25% in control subjects. No statistically significant differences were detected, indicating that the distribution of this genotype remains consistent across the groups (Fig. 4).

**Figure 5:**
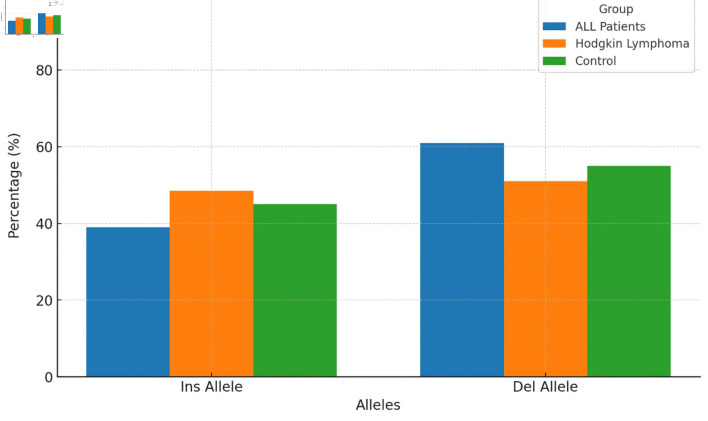
Allele frequencies of -94 ATTG polymorphism (rs28362491) across different groups. ALL: acute lymphoblastic leukemia.

The Ins allele frequencies were 56.5% in ALL patients, 50% in HL patients, and 42% in control subjects. The analysis did not reveal any statistically significant differences, suggesting a stable presence of the Ins allele across the groups. The Del allele displayed a frequency of 43.5% in ALL patients, 50% in HL, and 58% in control subjects. Like the Ins allele, no statistically significant variations were noted, indicating that the Del allele is consistently distributed across different health statuses and diseases (Fig. 5).

The observed genotype frequencies imply a possible association between the Ins/Ins and Ins/Del genotypes and ALL compared to control subjects, potentially indicating a genetic predisposition or resistance related to these specific genotypes. The significant differences in the Ins/Ins and Ins/Del genotypes between ALL patients and control subjects underscore the importance of these genotypes in the context of ALL. The lack of significant differences in the allele frequencies across the groups implies a general stability of these alleles in the population, regardless of health status or disease. This finding suggests that while specific genotypes might be associated with disease, the overall allele distribution remains consistent.

In conclusion, this analysis of the -94 ATTG polymorphism (rs28362491) provides crucial insights into the genetic factors that may influence the susceptibility to or progression of ALL and HL. The statistically significant differences in genotype frequencies between ALL patients and control subjects suggest probable genetic markers for ALL susceptibility.

### Association between -94 ATTG polymorphism genotypes and miR-206 expression levels in ALL patients

**Figure 6:**
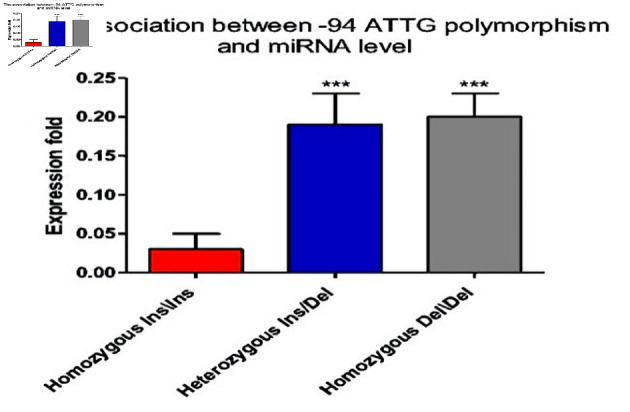
Association between -94 ATTG polymorphism genotypes and miR-206 expression levels in ALL patients. ALL: acute lymphoblastic leukemia; miRNA: microRNA.

This study further investigated the potential influence of the -94 ATTG polymorphism (rs28362491) on miR-206 expression across different genotypes in ALL patients. The genotypes examined were homozygous Ins/Ins, heterozygous Ins/Del, and homozygous Del/Del. miR-206 expression levels in individuals with the homozygous Ins/Ins genotype were 0.01 ± 0.05. For those with the heterozygous Ins/Del genotype, the expression level was similarly low at 0.01 ± 0.04. Individuals with the homozygous Del/Del genotype also exhibited a low level of miR-206 expression, recorded at 0.01 ± 0.02 (Fig. 6).

Statistical analysis of miR-206 expression levels across these genotypes showed no statistically significant differences. The comparisons of P values to the Ins/Ins genotype did not yield statistical significance for either the Ins/Del or Del/Del genotypes. Similarly, there were no statistically significant differences observed in the comparisons between the Ins/Del and Del/Del genotypes. The uniformly low expression levels of miR-206 across all genotypes suggest that the -94 ATTG polymorphism does not significantly influence miR-206 expression in the plasma of individuals. This finding indicates that miR-206 regulation may be independent of this specific genetic variation under the conditions studied.

### Association between -94 ATTG polymorphism genotypes and WBC levels in ALL patients

**Figure 7:**
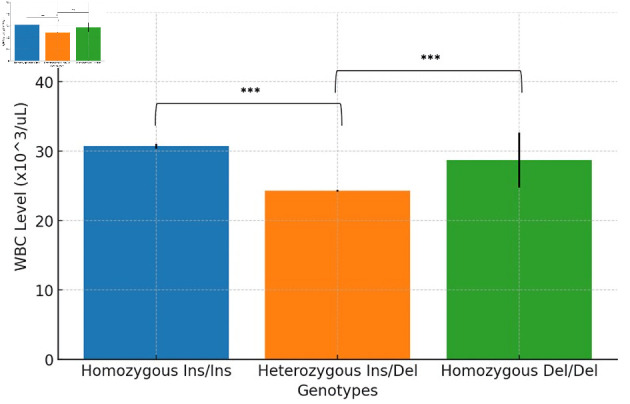
Association between -94 ATTG polymorphism genotypes and WBC levels in ALL patients. WBC: white blood cell; ALL: acute lymphoblastic leukemia.

We evaluated the influence of the -94 ATTG polymorphism (rs28362491) genotypes on WBC levels among ALL patients. The analysis focused on three genotypes: homozygous Ins/Ins, heterozygous Ins/Del, and homozygous Del/Del. The WBC level analysis revealed that patients with the homozygous Ins/Ins genotype presented with the highest mean WBC level at 30.7 ± 0.37 × 10^3^/µL. Patients with the heterozygous Ins/Del genotype exhibited a lower mean WBC level of 24.3 ± 0.11 × 10^3^/µL. This group showed a statistically significant decrease in WBC levels compared to the homozygous Ins/Ins genotype. Meanwhile, patients with the homozygous Del/Del genotype had a mean WBC level of 28.7 ± 3.98 × 10^3^/µL (Fig. 7). Although this genotype’s WBC level was higher than that of the heterozygous Ins/Del, it did not show a statistically significant difference when compared directly with the heterozygous Ins/Del genotype. However, a significant difference was noted compared to the homozygous Ins/Ins genotype. The WBC levels varied significantly across genotypes within ALL patients, with the homozygous Ins/Ins genotype associated with the highest WBC counts. This suggests a possible genetic influence of the -94 ATTG polymorphism on the immune response or leukemia progression in these patients. The lower WBC levels in patients with the heterozygous Ins/Del genotype could indicate a differential impact of the mixed genetic background on leukocyte proliferation or survival.

### Association between -94 ATTG polymorphism genotypes and disease status in ALL patients

**Figure 8:**
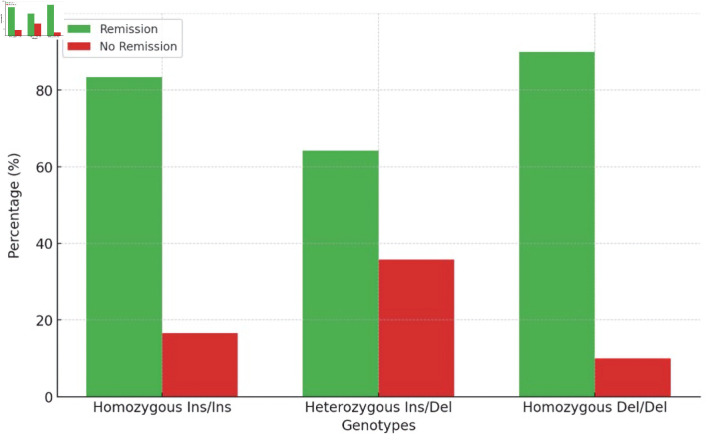
Association between -94 ATTG polymorphism genotypes disease status in ALL patients. ALL: acute lymphoblastic leukemia

This study evaluated the influence of the -94 ATTG polymorphism (rs28362491) genotypes on disease remission rates among ALL patients. The analysis focused on three distinct genotypes: homozygous Ins/Ins, heterozygous Ins/Del, and homozygous Del/Del. Patients with the homozygous Ins/Ins genotype exhibited a notable complete remission (CR) rate, with 83.3% achieving CR, contrasting with a 16.6% non-remission rate (NoCR). This suggests that the homozygous Ins/Ins genotype may be associated with a favorable response to treatment and a higher likelihood of achieving CR (Fig. 8). Patients with the heterozygous Ins/Del genotype showed a lower CR rate of 64.2%, with 35.7% of patients failing to achieve CR. Statistical analysis indicated that the difference in remission rates between this genotype and the homozygous Ins/Ins genotype was not statistically significant. This suggests that while the heterozygous Ins/Del genotype may be associated with a lower CR rate, the difference is not pronounced enough to be statistically significant (Fig. 8). The homozygous Del/Del genotype presented the highest CR rate at 90%, with only 10% of patients not achieving CR. Like the heterozygous Ins/Del genotype, comparisons to other genotypes did not reveal statistically significant differences (Fig. 8). This high CR rate suggests a potential beneficial effect of the homozygous Del/Del genotype on treatment outcomes, though the lack of statistical significance indicates that this effect may not be robust across all patients.

The study indicates trends in CR rates across various genotypes of the -94 ATTG polymorphism, although statistical significance was not observed. The high CR rates associated with both homozygous Ins/Ins and homozygous Del/Del genotypes may indicate a genetic influence on treatment response, although further studies with larger sample sizes are needed to confirm these trends and determine their clinical relevance.

### Association between miR-206 and disease status in ALL patients

**Figure 9:**
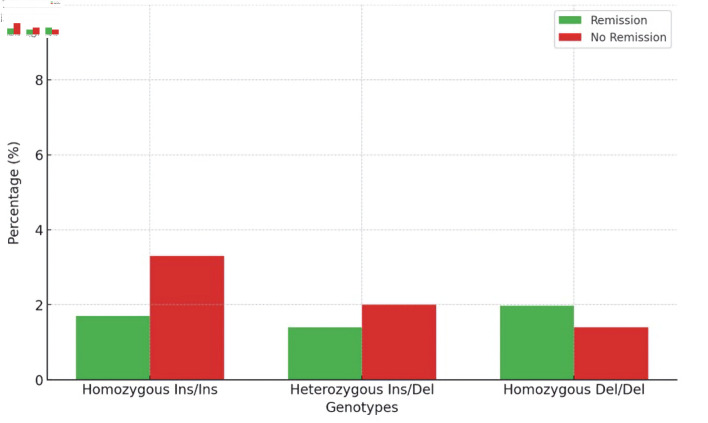
Association between miR-206 and disease status in ALL patients. ALL: acute lymphoblastic leukemia.

This study evaluated the correlation between miR-206 levels and disease remission among ALL patients, stratified by genotypes of the -94 ATTG polymorphism (rs28362491). The analysis included three genotypes: homozygous Ins/Ins, heterozygous Ins/Del, and homozygous Del/Del (Fig. 9). miR-206 levels in patients with the homozygous Ins/Ins genotype were 1.7% in those who achieved CR, compared to 3.3% in those who did not achieve remission. For the heterozygous Ins/Del genotype, miR-206 levels were 1.4% in patients in CR, compared to 2% in patients not achieving remission. Patients with the homozygous Del/Del genotype showed miR-206 levels of 1.97% in CR and 1.4% in non-remission. No statistically significant differences in remission rates were observed between heterozygous Ins/Del and homozygous Del/Del genotypes compared to the homozygous Ins/Ins genotype (P = 0.9086). The results indicate that while there are observed differences in miR-206 levels among the genotypes, these differences do not translate into statistically significant variations in remission rates among the genotypes studied. Each genotype shows a unique pattern of miR-206 levels in remission versus no-remission states. However, statistically, the differences between genotypes in terms of remission do not reach statistical significance.

## Discussion

miRNAs are a class of naturally occurring short non-coding RNAs, typically 21–22 nucleotides in length. These molecules play a crucial role in post-transcriptional gene silencing. A single miRNA can influence the expression of thousands of miRNAs and their corresponding target gene [16, 17]. Generally, miRNAs bind to the 3′ untranslated region (3′UTR) of target miRNAs to either inhibit translation or promote transcript degradation. The essential seed region of miRNAs, which is responsible for their target recognition, is located within nucleotides 2–7 of the 5′UTR. miRNAs are involved in various critical regulatory functions, including cell growth, development, and differentiation. Dysregulation of miRNAs has been linked to numerous human diseases, particularly cancers.

miR-206 is part of the miR-1 family and is encoded by a gene located between the *IL-17* and *PKHD1* genes in humans, specifically at the cytogenetic band 6p12.2. Several studies have examined the expression of miR-206 in various types of cancers, elucidating its molecular mechanisms in carcinogenesis. In bladder cancer, an experiment revealed that long non-coding RNA (lncRNA) RMRP (RNA component of mitochondrial RNA processing endoribonuclease) is overexpressed in tumor tissues compared to adjacent normal tissues, as measured by qRT-PCR. Functional assays, including MTT (3-(4,5-dimethylthiazol-2-yl)-2,5-diphenyltetrazolium bromide) and Transwell assays, demonstrated that RMRP promotes cell proliferation, migration, and invasiveness through the regulation of miR-206. This conclusion was supported by luciferase assays showing the binding interaction between miR-206 and RMRP [18]. In breast cancer, miR-206 acts as a tumor suppressor, potentially by downregulating PFKFB3. Studies have shown that miR-206 expression is reduced in estrogen receptor α (ERα)-positive breast cancer cells in a dose-dependent manner by 17β-estradiol. Overexpression of miR-206 can inhibit the production of fructose-2,6-bisphosphate, decrease lactate synthesis, and limit the migratory and proliferative potential of breast cancer cells [19]. Additionally, the downregulation of miR-206 has been linked to larger tumor size and advanced clinical stages in breast cancer [20]. Overexpression of miR-206 in MCF-7 cells has been shown to suppress cell growth by hindering the G1/S transition, mediated through the suppression of cyclin D2 expression. In breast cancer tissues, miR-206 and cyclin D2 levels have consistently been found to be inversely correlated. Furthermore, miR-206 expression is reduced in ERα-positive breast tumors [20]. miR-206 has also been found to be downregulated in cervical cancer tissues [21], while its target gene *c-Met* is upregulated, as evidenced by qRT-PCR and immunohistochemistry assays [22]. Additionally, the downregulation of miR-206 in cervical cancer is linked to lymph node metastasis, advanced stage, and higher histological grade, indicating its role in the metastasis and progression of this cancer. miR-206 has been identified as an independent prognostic marker for overall survival in cervical cancer patients [22].

Our findings align with these previous studies, reinforcing the role of miR-206 as a tumor suppressor. The significant reduction of miR-206 expression in ALL and HL patients suggests that its downregulation may contribute to the pathogenesis and progression of these hematologic malignancies. Future research should focus on elucidating the precise mechanisms through which miR-206 exerts its tumor-suppressive effects and exploring its potential as a therapeutic target in these cancers. The marked downregulation of miR-206 in ALL and HL patients compared to healthy controls highlights its potential as a tumor suppressor. This study contributes to the growing body of evidence supporting miR-206’s involvement in cancer pathogenesis and highlights the need for further investigation into its therapeutic potential.

The role of miR-206 in inhibiting tumor growth and progression is mediated through multiple molecular mechanisms, involving the regulation of target genes and signaling pathways that are crucial for cancer cell survival, proliferation, and metastasis. One of the primary mechanisms by which miR-206 exerts its tumor suppressor function is through the inhibition of oncogenes. For instance, in cervical cancer, miR-206 downregulates the expression of c-Met, a receptor tyrosine kinase that promotes cell proliferation, migration, and invasion. By targeting c-Met, miR-206 inhibits these oncogenic processes, thereby reducing the metastatic potential of cancer cells (Sun et al, 2015 [22]). Similarly, in breast cancer, miR-206 targets and suppresses cyclin D2, a protein essential for the transition from the G1 to the S phase of the cell cycle. By inhibiting cyclin D2, miR-206 impedes cell cycle progression, leading to reduced cell proliferation and tumor growth [20].

miR-206 also modulates key signaling pathways involved in cancer cell growth and survival. The PI3K/AKT signaling pathway is one such pathway that miR-206 interferes with, reducing the activation of downstream effectors involved in promoting cell proliferation and inhibiting apoptosis. Additionally, miR-206 hampers the Wnt/β-catenin signaling pathway by targeting key molecules, thereby reducing the transcriptional activity of β-catenin, which is responsible for the expression of genes involved in cell proliferation and survival [22, 23]. Beyond its role in tumor growth suppression, miR-206 is crucial in preventing metastasis. Its ability to inhibit key molecules and signaling pathways involved in epithelial-mesenchymal transition (EMT), invasion, and migration makes it a critical regulator of cancer metastasis. By downregulating *c-Met* and other EMT-related genes, miR-206 reduces the invasive and migratory capabilities of cancer cells, thus limiting their metastatic potential [24].

In the context of NF-κB1, the findings from our study provide valuable insights into the role of the NF-κB1 (rs28362491) polymorphism in hematologic malignancies, specifically ALL and HL. The NF-κB1 pathway is crucial in regulating immune response, inflammation, and cell survival, and its dysregulation has been implicated in various cancers [25]. Our analysis revealed distinct genotypic distributions of the NF-κB1 (rs28362491) polymorphism among ALL patients, HL patients, and healthy controls. The results showed a higher prevalence of the Ins/Ins genotype in ALL patients compared to controls and a lower prevalence of the Ins/Del genotype in ALL patients. No significant differences in genotype distributions were found between ALL and HL patients or between HL patients and controls. These findings suggest that the Ins/Ins genotype of the NF-κB1 (rs28362491) polymorphism may be associated with an increased risk of ALL, while the Ins/Del genotype may have a protective effect. The absence of significant differences between HL patients, ALL patients, and controls implies that this polymorphism might not significantly influence susceptibility to HL.

Comparing our results with existing literature reveals both consistencies and discrepancies. In a study by de Jonge et al (2009), the NF-κB1 (rs28362491) polymorphism was evaluated in pediatric ALL patients, and a significant association was found between the Ins/Ins genotype and increased risk of ALL, which aligns with our findings [26]. The Ins/Ins genotype is thought to result in higher NF-κB1 expression, leading to increased NF-κB1 activity and enhanced survival and proliferation of leukemic cells. Conversely, Luo et al (2022) did not find a significant association between the NF-κB1 (rs28362491) polymorphism and ALL risk in a Chinese population, highlighting potential ethnic differences in genetic susceptibility [27]. These mixed results underscore the need for larger, multicenter studies to clarify the role of this polymorphism in ALL.

In HL, the literature on the NF-κB1 (rs28362491) polymorphism is more limited. Pakjoo et al (2024) investigated this polymorphism in Indian HL patients and found no significant association between the NF-κB1 (rs28362491) genotypes and HL risk, which is consistent with our findings [28]. This suggests that the NF-κB1 (rs28362491) polymorphism may not be a major factor in HL susceptibility, or its impact may vary by population. The role of NF-κB1 polymorphisms in other hematologic malignancies has also shown varying associations. For example, Hill et al (2006) [29] reported an association between the Ins/Ins genotype and increased risk of non-Hodgkin lymphoma, while Albensi (2019) [5] found no significant association in multiple myeloma patients [26].

The NF-κB1 (rs28362491) polymorphism impacts NF-κB1 mRNA and protein levels by altering transcription factor binding to the NF-κB1 promoter. The Ins allele is associated with higher NF-κB1 expression, leading to increased NF-κB activity. Enhanced NF-κB activity can promote cell survival, proliferation, and resistance to apoptosis, contributing to cancer development and progression. In ALL, the higher prevalence of the Ins/Ins genotype may result in increased NF-κB activity, providing a survival advantage to leukemic cells. Clinically, current findings suggest that the NF-κB1 (rs28362491) polymorphism, particularly the Ins/Ins genotype, may serve as a genetic marker for ALL susceptibility. Identifying individuals with this genotype could help in early detection and personalized treatment strategies. However, the lack of significant association in Hodgkin lymphoma indicates that this polymorphism may not be a universal marker for all hematologic malignancies.

The study examined the genotype and allele frequencies of the -94 ATTG polymorphism (rs28362491) in three groups. Our results revealed that the homozygous Ins/Ins genotype was most prevalent in ALL patients (41.3%), followed by HL patients (28.5%), and was least common in control subjects (12%). There was a statistically significant difference between ALL patients and control subjects (P = 0.0048), suggesting that the Ins/Ins genotype may be more frequently associated with ALL and could represent a potential genetic risk factor for this disease. This finding aligns with previous studies that have identified the Ins/Ins genotype as a contributor to increased NF-κB1 expression, leading to heightened NF-κB activity and enhanced survival and proliferation of leukemic cells. For instance, Canevarolo et al (2023) reported a similar association between the Ins/Ins genotype and increased ALL risk, reinforcing the idea that this genotype contributes to the pathogenesis of ALL [30].

Conversely, the heterozygous Ins/Del genotype was most prevalent in the control group (60%), followed by HL patients (42.8%), and was least common in ALL patients (30.4%). There is a statistically significant difference between ALL patients and control subjects suggesting that the Ins/Del genotype may confer a protective effect against ALL or indicating different genetic predispositions. The higher frequency of the Ins/Del genotype in the control group suggests a balanced genetic variability that may reduce the susceptibility to ALL. This observation is supported by literature that often highlights heterozygosity as a factor that can contribute to genetic resilience against certain diseases. For example, Hou et al (2021) found that the Ins/Del genotype was not significantly associated with ALL risk in a Chinese population, which may reflect the protective nature of heterozygosity in diverse genetic backgrounds [31].

The homozygous Del/Del genotype showed consistent frequencies across the various groups studied, with 28.2% in ALL patients, 28.5% in HL patients, and 25% in control subjects. The lack of statistically significant differences in the Del/Del genotype distribution suggests that this genotype does not vary significantly between the groups and may not play a distinct role in the susceptibility to ALL or HL. This finding is consistent with other studies that have reported no strong associations between the Del/Del genotype and cancer risk, indicating that its impact may be neutral or minimal in the context of these hematologic malignancies [32].

In HL, the distribution of the -94 ATTG polymorphism genotypes did not show statistically significant differences compared to controls or ALL patients, indicating that this polymorphism may not be a major determinant of genetic susceptibility in Hodgkin lymphoma. This is in line with the study by Fu et al (2017), who found no statistically significant association between the NF-κB1 (rs28362491) genotypes and Hodgkin lymphoma risk, suggesting that other genetic or environmental factors may play a more critical role in the disease’s pathogenesis [33].

Overall, our findings contribute to the growing body of evidence regarding the role of the -94 ATTG polymorphism (rs28362491) in hematologic malignancies. The statistically significant association of the Ins/Ins genotype with ALL highlights its potential as a genetic marker for susceptibility, while the protective effect suggested by the Ins/Del genotype in the control group warrants further investigation. These insights underscore the complexity of genetic factors in cancer development and the need for continued research to elucidate the mechanisms underlying these associations.

This study also evaluated the correlation between miR-206 levels and disease status among ALL patients, stratified by genotypes of the -94 ATTG polymorphism (rs28362491). Our results showed no statistically significant differences in miR-206 levels between patients in CR and those not in remission across the three genotypes (homozygous Ins/Ins, heterozygous Ins/Del, and homozygous Del/Del). Specifically, miR-206 levels were slightly lower in CR for the Ins/Ins and Ins/Del genotypes but did not differ significantly (P = 0.9086). Comparing these results with existing literature reveals both consistencies and discrepancies. Studies have shown that miR-206 acts as a tumor suppressor, and its downregulation is associated with various cancers, including breast and bladder cancers. In breast cancer, for example, miR-206 targets and downregulates oncogenes such as *PFKFB3*, reducing tumor cell proliferation and invasion [19, 20].

The role of miR-206 in leukemia, particularly in the context of genetic polymorphisms, is less well-documented. Our study results suggest that miR-206 levels do not significantly differ between remission and non-remission groups across the -94 ATTG polymorphism genotypes, indicating that miR-206 may not play a crucial role in the remission status of ALL patients. In hematologic malignancies, miRNA may have a different role compared to solid tumors, suggesting a divergence in miRNA function between these two types of cancers.

The study had several limitations, including the small and heterogenous sample size in each group and the use of samples derived from different sources, such as tissue and blood, which may have affected the consistency of RNA and DNA extraction and measurements.

### Conclusions

This study investigated the correlation between miR-206 levels and disease remission in ALL patients stratified by genotypes of the -94 ATTG polymorphism (rs28362491). The study results showed no significant differences in miR-206 across different genotypes, no significant genotype effect on remission status in ALL patients, and both abnormalities could serve as biomarkers. These results align with earlier studies emphasizing the intricate and context-dependent functions of miRNA and genetic polymorphisms in cancer. Additionally, our study aligns with the literature indicating that, although miR-206 and NF-κB1 polymorphisms are significant in other cancers, their roles in ALL and HL may be less pronounced.

From a future perspective, future research should consider the following directions: 1) larger and diverse cohorts to validate the findings and understand the genetic variability across different populations; 2) functional studies investigating the functional mechanisms underlying the interaction between miR-206 and NF-κB1 pathways in ALL; 3) longitudinal studies monitoring changes in miR-206 expression and NF-kB1 polymorphism genotypes longitudinally in ALL patients; 4) exploration of therapeutic potential of miR-206 modulation in ALL; 5) broader genetic analysis including other polymorphisms within the NF-κB pathway and related signaling networks; and 6) comparative studies evaluating the roles of miR-206 and NF-κB polymorphisms in ALL relative to other hematologic malignancies and solid tumors.

## Supplementary Material

Suppl 1 Tissue and blood sample collection and preparation, RNA and DNA extraction and quantification.

## Data Availability

The authors declare that data supporting the findings of this study are available within the article

## Abbreviations

ALL: acute lymphoblastic leukemia
AML: acute myeloid leukemia
KHCC: King Hussein Cancer Center
miRNAs: microRNAs
NF-κB: nuclear factor kappa B
PDRC: Pharmacological and Diagnostic Research Center
RMRP: RNA component of mitochondrial RNA processing endoribonuclease
qRT-PCR: quantitative real-time polymerase chain reaction
WBC: white blood cell
