# Supplementary material for: The Association Between Potential Nuclear Factor-Kappa B1 Gene Polymorphism rs28362491 and miR-206 Level in Patients With Acute Lymphoblastic Leukemia
Source: World J Oncol. 2026 Jun 25;17(4):463–76. doi: 10.14740/wjon2762 (PMC13375431; doi:10.14740/wjon2762)
Supplement: Suppl 1 — Tissue and blood sample collection and preparation, RNA and DNA extraction and quantification. [file wjon-17-04-463-s001.docx]

# **Suppl 1.** Tissue, blood sample collection and preparation, RNA and DNA extraction and quantification

## Tissue sample collection

## Tissue samples were collected from patients diagnosed with ALL and HL at the haematology and oncology clinics of KHCC. These samples were already stored at KHCC, and the study utilized pre-existing specimens. Biopsy samples were obtained from affected lymphnodes or bone marrow, depending on the type and location of the malignancy. The samples were initially preserved in sterile containers with 10% neutral buffered formalin, ensuring the preservation of tissue morphology and cellular integrity. These specimens were then transported to the laboratory department for further processing.

## Tissue sample preparation

The tissue samples underwent a paraffin-embedding process, a widely used technique in clinical and research settings to preserve tissue in a formalin-fixed, paraffin-embedded (FFPE) block. This process involves fixation with formalin, followed by dehydration through a graded series of ethanol solutions, ranging from 70% to 100%, to remove water content and prepare the tissues for paraffin embedding. The dehydrated tissues were then treated with xylene, a clearing agent, to render them transparent and facilitate the infiltration of paraffin. Subsequently, the cleared tissues were infiltrated with molten paraffin wax at 60°C. The tissues were oriented in embedding Mold and allowed to solidify, forming paraffin blocks, which provided a stable medium for sectioning. The paraffin-embedded tissues were stored at temperatures not exceeding 27°C (80°F) in plastic zip-lock bags, labeled with the LDMS barcode, per local guidelines. These paraffin blocks are retained at KHCC for up to 15 years.

To prepare the sections, the paraffin-embedded tissue blocks were initially placed on ice before sectioning. This process supports the harder elements within the tissue, allowing for thinner sections to be obtained, and the slight moisture from the melting ice facilitates easier cutting. A water bath filled with ultrapure water was heated to between 40-45°C. The microtome blade was secured in its holder, and the clearance angle was set according to the manufacturer’s guidelines, typically between 1° and 5° for Leica blades.

The paraffin block was oriented so that the blade could cut straight across it. The blade was carefully brought near the block to slice a few initial thin sections, ensuring correct alignment. Adjustments were made as needed. The block was trimmed at a thickness of 10-30 µm to expose an adequate surface of the tissue, from which representative sections were then cut at about 4-5 µm thickness. The first few sections, likely containing holes from the trimming, were usually discarded. Once the sections were cut, they were picked up with tweezers and floated on the surface of the heated water in the bath to flatten. Using the tweezers, the sections were separated and then carefully transferred onto microscope slides. The slides were then placed upright in a slide rack and dried overnight in an oven set to 37°C, ensuring the sections were ready for subsequent analysis.

Blood collection and plasma preparation

## Blood collection

Blood samples were collected from the healthy control group. The collection process followed standard clinical procedures to ensure the integrity and quality of the samples. Each participant's venipuncture was performed using a sterile technique with a 21-gauge needle, and 5 mL of blood was drawn into EDTA tubes to prevent coagulation.

The blood samples were then gently inverted several times to mix the anticoagulant thoroughly with the blood, preventing clot formation. The collected samples were labeled with unique identifiers and immediately transported to the laboratory for further processing.

## Plasma preparation

Upon arrival at the laboratory, the blood samples were centrifuged at 5000 rpm for 15 minutes at 4°C to separate the plasma from the cellular components. The plasma, being the supernatant, was carefully aspirated using a pipette and transferred into new sterile tubes. Care was taken to avoid disturbing the buffy coat layer to prevent contamination with white blood cells. The plasma samples were then aliquoted into smaller volumes to minimize freeze-thaw cycles, which can degrade the quality of the plasma. Each aliquot was labeled with the corresponding unique identifier and stored at -80°C until further analysis. Before conducting any analysis, the plasma samples were thawed on ice to preserve their integrity. The thawed samples were then gently mixed to ensure homogeneity. These prepared plasma samples were used for subsequent biochemical and molecular analyses, providing critical data for the study.

To prepare the samples for miRNA extraction, excess paraffin was first trimmed from the sample block using a scalpel. Sections ranging from 5–20 µm thick were then cut from the paraffin-embedded tissue blocks. The first 2–3 sections, which may have been exposed to air, were discarded to avoid contamination. The selected sections were immediately placed into a microcentrifuge tube, and the lid was securely closed.

To deparaffinize the tissue sections, 200 μL of Deparaffinization solution was added to each tube. The tubes were vigorously vortexed for 10 seconds and briefly centrifuged to settle the sample to the bottom. The tubes were then incubated at 56°C for 3 minutes and allowed to cool to room temperature. Following the initial incubation, 150 μL of buffer PKD was added to each tube, and the samples were mixed by vortexing. They were then centrifuged for 1 minute at 10,000 rpm. After centrifugation, 10 μL of proteinase K was added to the lower, clear phase of the sample.

The samples were gently mixed by pipetting up and down and incubated first at 56°C for 15 minutes and subsequently at 80°C for another 15 minutes. During these incubations, the samples were briefly vortexed every 3–5 minutes if a heating block without a shaking function was used. If only one heating block was available, the samples were left at room temperature after the 56°C incubation until the block reached 80°C. This incubation step at 80°C was critical for optimal RNA performance in downstream applications, such as real-time RT-PCR, as it partially reverses the formaldehyde modification of the nucleic acids.

The lower, clear phase was then transferred to a new 2 mL microcentrifuge tube. If more than two sections were being processed, a larger tube was required. The transferred samples were placed on ice for 3 minutes and then centrifuged for 15 minutes at 20,000 rpm. The supernatant was carefully transferred to another new microcentrifuge tube, ensuring not disturb the pellet containing insoluble tissue debris. To the supernatant, DNase Booster Buffer, equivalent to a tenth of the total sample volume, and 10 μL DNase I stock solution were added. The mixture was gently inverted to mix and briefly centrifuged to collect any residual liquid from the sides of the tube. The samples were incubated at room temperature for 15 minutes to ensure complete digestion of any contaminating DNA.

Next, 500 μL of Buffer RBC was added to the samples to adjust the binding conditions, and the lysate was mixed thoroughly. Subsequently, 1750 μL of 100% ethanol was added to the samples, which were then mixed well by pipetting. The samples, including any precipitate that might have formed, were transferred in aliquots of 700 μL to a RNeasy MinElute spin column placed in a 2 mL collection tube. The lids were closed gently, and the columns were centrifuged for 15 seconds at 10000 rpm. The flow-through was discarded, and the collection tube was reused for subsequent steps.

After all the samples had passed through the column, 500 μL of Buffer RPE was added to the RNeasy MinElute spin column, and it was centrifuged for 15 seconds at 10000 rpm. The flow-through was again discarded, and the process was repeated with an additional 500 μL of Buffer RPE, followed by centrifugation for 2 minutes at the same speed to thoroughly wash the spin column membrane. After centrifugation, the collection tube with the flow-through was discarded. The RNeasy MinElute spin column was then placed in a new 2 mL collection tube, the lid of the column was opened, and it was centrifuged at full speed for 5 minutes to ensure the spin column membrane was completely dry, as residual ethanol could interfere with downstream reactions. The dry spin column was then placed in a new 1.5 mL collection tube, and 14 μLof RNase-free water was added directly to the centre of the spin column membrane. The column was centrifuged for 1 minute at full speed to elute the RNA. The eluted RNA was quantified using spectrophotometry to assess its concentration and purity, and its integrity was evaluated using agarose gel electrophoresis. These RNA samples were then stored at -80°C until further gene expression analysis.

To prepare the plasma samples for miRNA extraction, the plasma was first thawed on ice and gently mixed to ensure homogeneity. miRNA was extracted using a commercial miRNA extraction kit designed for plasma samples. Initially, 200 μL of plasma was mixed with 1 mL of QIAzol Lysis Reagent in a 2 mL microcentrifuge tube. The mixture was vortexed for 30 seconds to ensure thorough mixing. The tubes were then incubated at room temperature for 5 minutes to permit complete dissociation of nucleoprotein complexes. Following incubation, 200 μL of chloroform was added to each tube, and the tubes were shaken vigorously for 15 seconds. The samples were incubated at room temperature for another 3 minutes. The mixture was then centrifuged at 10,000 rpm for 15 minutes at 4°C. After centrifugation, the mixture separated into a lower red, phenol-chloroform phase, an interphase, and a colourless upper aqueous phase. The upper aqueous phase, which contains the miRNA, was carefully transferred to a new microcentrifuge tube, ensuring not disturb the interphase.

To precipitate the miRNA, 500 μL of 100% isopropanol was added to the aqueous phase, and the tubes were incubated at room temperature for 10 minutes. The samples were then centrifuged at 12,000 rpm for 10 minutes at 4°C. The supernatant was carefully removed, and the miRNA pellet was washed with 1 mL of 75% ethanol. The samples were vortexed briefly and centrifuged at 10,000 rpm for 5 minutes at 4°C. The ethanol was then carefully removed, and the miRNA pellet was air-dried for 5-10 minutes. The dried miRNA pellet was resuspended in 20-30 μL of RNase-free water and incubated for 10 minutes at 55°C to facilitate dissolution. The concentration and purity of the extracted miRNA were assessed using spectrophotometry, and its integrity was evaluated using agarose gel electrophoresis. These miRNA samples were then stored at -80°C until further gene expression analysis.

# Synthesis of complementary deoxyribonucleic acid (cDNA)

To synthesize cDNA from the extracted miRNA, the following protocol was employed. First, the template RNA and 5× miRCURY RT Reaction Buffer were thawed on ice, while RNase-free water was thawed at room temperature (15–25°C). Immediately before use, the 10× miRCURY RT Enzyme was removed from the freezer. Each solution was mixed by flicking the tubes and centrifuged briefly to collect residual liquid from the sides of the tubes before keeping them on ice.

Next, the UniSp6 RNA spike-in was resuspended by adding 80 μL of nuclease-free water to the tube. The mixture was vortexed and spun down, then left on ice for 20–30 minutes to completely dissolve the RNA spike-in. After this period, the solution was mixed again by vertexing and spun down, with the resuspended UniSp6 RNA spike-in stored in aliquots at –30 to –15°C. Subsequently, each template RNA was adjusted to a concentration of 5 ng/μL. For RNA isolated from serum or plasma, the amount of RNA equivalent to that isolated from 16 μL of the sample was calculated for a 20 μL reverse transcription (RT) reaction. For instance, for a sample isolated from 200 μL of plasma and eluted in 50 μL, 4 μL of the eluate was used.

The reverse transcription master mix was prepared on ice according to the specified proportions. The mixture was kept on ice, and if more than one reaction was set up, a master mix with a volume 10% greater than required for the total number of reactions was prepared. The appropriate volume of the master mix was then distributed into individual tubes, followed by the addition of each RNA sample. The reaction mixtures were incubated for 60 minutes at 42°C to facilitate reverse transcription. Following this incubation, the reaction mixtures were incubated for 5 minutes at 95°C to inactivate the Reverse Transcriptase Enzyme. The reactions were immediately cooled to 4°C. If not used immediately, the reverse transcription reactions were stored at 4°C, with long-term storage at –30 to –15°C. The reverse transcription reactions were then placed on ice and proceeded directly to real-time PCR. Recommendations for dilutions in sterile water were followed according to the respective handbooks, depending on the downstream application. The miRCURY LNA SYBR® Green PCR Kit was recommended for real-time PCR. Detailed information on the use of the RNA Spike-in Kit and interpretation of real-time PCR results was referred to in the RNA Spike-in Kit for RT Quick-Start Protocol.

# Relative quantification of miRNA levels

Plasma and tissue levels of miRNA were quantified using quantitative real-time PCR (qRT-PCR) with Fast SYBR Green. The PCR master mix was prepared on ice according to the manufacturer’s instructions. This included the SYBR® Green Master Mix, the appropriate miRCURY LNA PCR primer set specific to the target miRNA, and the cDNA template. For each reaction, 10 µL of the SYBR® Green Master Mix was combined with 1 µL of the miRCURY LNA PCR primer set and 1 µL of cDNA template. RNase-free water was added to bring the total reaction volume to 20 µL. If multiple reactions were set up, a master mix with a volume 10% greater than required for the total number of reactions was prepared to ensure consistency across samples. The PCR reactions were set up in a 96-well plate, with each sample, including the no-template controls and reference genes, pipetted into individual wells. The plate was sealed and briefly centrifuged to ensure all liquid was collected at the bottom of the wells and to remove any air bubbles. The real-time PCR thermal cycler was programmed for 40 cycles with the following conditions: an initial activation step at 95°C for 2 minutes, denaturation at 95°C for 10 seconds, followed by annealing and extension at 56°C for 1 minute. The fluorescence data were collected at the end of each cycle during the annealing and extension step.

To ensure the accuracy and reliability of the quantification, all reactions were performed in triplicate. miRNA expression data were calculated using the Pfaffl method (Pfaffl, 2001), which accounts for varying efficiencies of target and reference genes. The relative quantification (Q) was determined using the following formula:

$$Relative Qncentration=\frac{\left( E_{target sample} \right)^{\Delta Ct\left( target sample \right)}}{\left( E_{reference sample} \right)^{\Delta Ct\left( reference sample \right)}}\ldots\ldots\ldots\ldots\ldots\ldots\ldots[1]$$

where:

$$\Delta Ct\left( \mathrm{reference} \right)= \mathrm{Ct}\left( reference gene in calibrator \right)- Ct\left( reference gene in test \right)$$

$$\Delta Ct\left( \mathrm{target} \right)=Ct\left( target gene in calibrator \right)-Ct\left( target gene in test \right)\Delta Ct\left( \mathrm{reference} \right)$$

The amplification efficiency (E) was calculated using the following formula:

$Efficiency= \frac{{10}^{-1}}{\mathrm{Slope}}$…………………. [2]

In cases where the amount of reference and target regions doubled each cycle, the efficiency of both target and reference genes was assumed to be 2, corresponding to a slope of -3.32. The relative quantification could then be simplified to:

$Relative Concentration= 2^{-\Delta\Delta Ct}$…………………… [3]

# DNA extraction for SNP of NF-κB

First, tissue samples were collected and placed in 1.5 mL microcentrifuge tubes. Each sample was finely minced and then homogenized in 200 µL of lysis buffer provided in the kit. Following homogenization, 20 µL of Proteinase K was added to each tube, and the samples were incubated at 56°C for 30 minutes to ensure complete digestion of proteins and lysis of the cells. The tubes were occasionally inverted to mix the contents during the incubation period. After incubation, 200 µL of Binding Buffer was added to each tube, and the mixture was vortexed for 15 seconds to ensure thorough mixing. The samples were then incubated at 70°C for 10 minutes. Next, 200 µL of 100% ethanol was added to each tube, and the mixture was vortexed again for 15 seconds.

The entire lysate was transferred to a G-spin™ column placed in a 2 mL collection tube. The columns were centrifuged at 8,000 *g* for 1 minute, and the flow-through was discarded. The columns were then washed with 500 µL of Wash Buffer 1 and centrifuged for 1 minute at 8,000 rpm. This step was repeated with Wash Buffer 2, followed by centrifugation at 12,000 rpm for 3 minutes to remove any residual ethanol. The columns were then transferred to new 1.5 mL microcentrifuge tubes. To elute the DNA, 200 µL of Elution Buffer was added directly onto the column matrix, and the columns were incubated at room temperature for 1 minute. The columns were then centrifuged at 8,000 rpm for 1 minute to collect the purified DNA. The eluted DNA was quantified using spectrophotometry and stored at -20°C until further analysis.

For DNA extraction from plasma, 200 µL of plasma was used. The plasma samples were mixed with 200 µL of lysis buffer and 20 µL of Proteinase K, followed by incubation at 56°C for 30 minutes. During this incubation, the tubes were inverted occasionally to mix the contents. After incubation, 200 µL of Binding Buffer was added to each tube, and the mixture was vortexed for 15 seconds. The samples were then incubated at 70°C for 10 minutes. Next, 200 µL of 100% ethanol was added to each tube, and the mixture was vortexed again for 15 seconds. The entire lysate was transferred to a G-spin™ column placed in a 2 mL collection tube. The columns were centrifuged at 8,000 rpm for 1 minute, and the flow-through was discarded. The columns were then washed with 500 µL of Wash Buffer 1 and centrifuged for 1 minute at 8,000 *g*. This step was repeated with Wash Buffer 2, followed by centrifugation at 12,000 rpm for 3 minutes to remove any residual ethanol. The columns were then transferred to new 1.5 mL microcentrifuge tubes. To elute the DNA, 200 µL of Elution Buffer was added directly onto the column matrix, and the columns were incubated at room temperature for 1 minute. The columns were then centrifuged at 8,000 rpm for 1 minute to collect the purified DNA. The eluted DNA was quantified using spectrophotometry and stored at -20°C until further analysis.

# 1:10 Quantitative PCR (qPCR) for NF-kB

The NF-kB polymorphism (rs28362491; −94 ins/ Del CGTGCTGCCTGCGTTCCCCGACC [ATTG/-] ATTGGGCCCGGCAGGCGCTTCCTG) was analyzed using the TaqMan SNP Genotyping Assays. This assay utilizes two dye-labeled probes, VIC and FAM, along with two target-specific primers. The forward primer sequence was TGG GCA CAA GTC GTT TAT G, and the reverse primer sequence was CTG GAG CCG GTA GGG AAG.

For the qPCR setup, the following procedure was followed. DNA extracted from tissue and plasma samples was first quantified and diluted to the appropriate concentration. The reaction mix was prepared by combining the TaqMan Genotyping Master Mix, the two primers, and the VIC and FAM-labeled probes. Specifically, the reaction mix contained the following components per 20 µL reaction volume: 10 µL of TaqMan Genotyping Master Mix, 1 µL of the forward primer (10 µM), 1 µL of the reverse primer (10 µM), 0.5 µL of the VIC probe, 0.5 µL of the FAM probe, and 7 µL of nuclease-free water. Finally, 1 µL of the DNA template was added to each reaction well.

The reactions were set up in a 96-well plate, and each sample, including the no-template controls, was pipetted into individual wells. The plate was sealed and centrifuged briefly to ensure that all liquid was collected at the bottom of the wells and to remove any air bubbles. The qPCR amplification was carried out using a real-time PCR thermal cycler with the following cycling conditions: an initial denaturation step at 95°C for 10 minutes, followed by 40 cycles of 95°C for 15 seconds and 60°C for 1 minute. During the annealing and extension step, the fluorescence data were collected.

The VIC and FAM probes were designed to bind specifically to the insertion and deletion alleles of the NF-kB1 polymorphism, respectively. The fluorescence signals generated by these probes were measured at each cycle, and the allelic discrimination analysis was performed using the software associated with the real-time PCR system. The results were interpreted based on the relative fluorescence of the VIC and FAM dyes, allowing for the identification of the genotype of each sample.
